# A Criticism—“Prophylaxis,”

**Published:** 1906-10-15

**Authors:** C. M. Wright

**Affiliations:** Cincinnati, O.


### A CRITICISM—"PROPHYLAXIS."

BY C. M. WRIGHT, D.D.S., CINCINNATI, O.

MY DEAR MR. EDITOR:—I have been very much pleased with the DENTAL REGISTER since new blood has begun to be assimilated in its different tissues and organs, causing them to give clear evidence of a vigor which is satisfactory to the new reader, and of a regeneration which is refreshing to the old reader. Here is wishing you and the REGISTER success!

But—without opposition and friction which must be overcome, and without obstacles which must be put aside by strong arms, the muscles of those arms will atroply and become weak. No holding of them up by friends (Biblical) will keep the circulation busy, the nutrition active, and the muscle cells full and strong, and capable of vigorous contractions in defense. Therefor, in all kindness and love, with a smiling face and a cordial feeling, I want to attack you fiercely and savagely. I shall knock out your feet of clay upon which your metal body stands, and when you fall, shall leave you sticking in the mud (classic); unless you rise up on your brass stumps and fight me to a finish.

First: What do you mean by the term "Prophylaxis," which you fondle and kiss and dawdle before dental societies (notably at Columbus in December), and in "Editorial Events" I ask again and in a loud tone of voice, What do you *mean* by it, and why didn't you say so?

I listened in patience to the reading of your paper on prophylaxis at Columbus, and I thought something was the matter with my head. The term was so juggled with, that if it has a definite place and meaning among words, it certainly dodged its place and was false to its meaning in your paper. After the paper appeared in print, I read it, and prophylaxis was still hopping from one place to another in the medical lexicon.

Now, I take up the June REGISTER and "prophylaxis" greets me, and I find in the editorial, the same disposition to play hide and seek. My dear Mr. Editor, the word is a real thing. It isn't an *ignis fatuus*. In every dictionary general as well as medical, of old date as well as of new, "prophylaxis" stands out boldly, acknowledges its origin from the Greek, and claims to be related solely to the "guarding beforehand" the "hindering of," the "prevention or preventing from disease," and has no business whatever with the healing and curing of disease. Therapeutics has to do with healing and curing. Prophylaxis hasn't a thing to do, directly, with either healing or curing, because its office is, as its Greek root shows, *before* occurrence.

*Prophylaxis* deals with healthy tissue. *Therapeutics* treats *diseased* tissues and conditions.

Prophylaxis may be a very simple matter, and very easy to practice, as when you put a net over your head and hands, *to keep from being stung*, while you stir up a hive of bees.

Therapeutics extracts the sting (surgery) and mops amonia on the *inflammation* caused by the sting—*to cure it*—after having been stung. Prophylaxis may also be complex, wide reaching, and most difficult to accomplish. The regulating of the positions and occlusions of a badly arranged set of teeth, requiring years of time, ingenious surgery and mechanical contrivances involving subtle laws of physics may fall entirely within the province of prophylaxis. We may do all this for the purpose of preventing or hindering local disease of the teeth, of the gums, of the sockets, of contiguous sinuses, or for the prevention of systemic nutrition, respiration and tissue building. We do all this before any symptoms of local or systemic disease show at all. The careful mother, who, with a little rag polishes the twenty perfect teeth of her child in order that the child may escape dental caries and tooth ache in the future is practicing prophylaxis.

The dentist who sees the child and with explorers and magnifying mirrors fails to find *caries*, but *to prevent* a probable attack in the future, bathes the new, clean, healthy teeth with nitrate of silver, is practicing prophylaxis. Should he deem it wise to cap with gold every one of these teeth, or to cut and fill every natural and sound crevice or sulcus in these teeth for the purpose aforesaid of preventing, guarding against, hindering that which is not yet present but which experience and observation have taught him will occur in from 75 to 95 percent of cases, he is practicing prophylaxis—and *not* therapeutics.

Dental caries is a definite condition and stands alone. It is not erosion, or gingivitis, or stomachache, but just well marked out and clearly understood, and distinctly separated "dental caries." If this dental caries appears, and the dentist cuts it out of the tooth with excavators or burrs, or grind stones, he is practicing surgical therapeutics. He is curing the tooth of caries. When he fills the cavity which his therapeutic surgery has made, if his intention is to prevent a recurrence of the old disease, he is in the field of prophylaxis again, and here, I suppose is where we are liable to get our terms mixed.

To me it seems essential that scientific men, in writing and speaking and using such terms as we have here defined, should not loose sight of the fixed boundaries of definition. Remember the confusion to students of biology a quarter of a century ago, by the way the term "protoplasm" was mis-applied. "Germinal matter," "bioplasm," "reticulum," "spongioplasm," and any name that any writer might want to apply to the living part of a tissue was shuffled about in such a way that nobody understood anybody else in biology.

Mr. Editor, we may get into the same confusion if you are not more exact in sharply marking the borders of your term "prophylaxis."

Look at the way "*pyorrhea alveolaris*" has crept into the wrong pocket and the confused conceptions it has caused in diagnosis, etiology, therapeutics and prophylaxis.

Now, Mr. Editor, re-read your editorial in the June

REGISTER and see how wretchedly you have mixed sense and nonsense, if we have a clear idea of the meaning of "prophylaxis."

"We have sometimes been tempted to conclude that pyorrhea alveolaris was one of the diseases that would have to be classed as incurable. But since seeing the results 'of prophylaxis'—we have taken courage and will henceforth not dispair in *desperate* conditions."

That is, we will *cure* these desperate conditions of *disease* by preventing them from appearing after they have appeared.

Again:

"To see mouths in which the masticating functions have been almost wholly lost, with the most threatening complications *resulting, restored*" to function and health—by what? by prevention, by prophylactic measures. Dear Mr. Editor, the mixup is demoralizing to ones mental equilibrium. Do be careful. Of course, with your view, the trained nurse, the girl assistant, the denticure, could not do all that you consider as falling under the head of "prophylaxis." Your prophylaxis is without boundaries. It is an unfenced and unsurveyed wilderness. It includes everything in the practice of dentistry from polishing the teeth with a stick, the correction of irregularities, the *curing* of local and systemic, acute and chronic, contiguous and remote diseases, by medicine, surgery or mechanics, to the final regulation of the production of species and the extraction of imperfect specimens. Ach, Gott!

Dr. D. D. Smith has done, and is doing, a grand work in dentistry. His "prophylaxis" does not consist of the treating and curing of gingivitis, pyorrhea or pericemental abscesses, but of the "polishing" of every exposed surface of every healthy tooth, thereby changing its environment from a state of dangerous uncleanliness to one of *cleanliness*. He has shown that the patient cannot keep his own teeth clean, that any operator skilled in this art must do it for him *with great frequency*. That *clean* teeth will remain *free from disease*. This is all, and it is the basis of his prophylaxis. I always take off my hat and bow with respect and reverence to the apostle of modern conception of immaculate teeth.

I do not accept his pathology and bypathology, I mean the doctrine and definition of disease including its etiology.

Dr. Smith does not define "disease" as I should. He does not believe that inflammation is a *disease*. He is a gallant fighter, and I like to sit safely ensconsed in a proscenium box, and watch this magnificent gladiator doing his stunt in the arena. It is fine. And I applaud as vociferoulsy as you can, Mr. Editor, as the combat proceeds. "Hurrah for Smith, and prophylaxis!" Vive la conqueror!

